# Fixed-Dose Versus Weight-Adapted Immune Checkpoint Inhibitor Therapy in Melanoma: A Retrospective Monocentric Analysis of Efficacy and Immune-Related Adverse Events

**DOI:** 10.3390/cancers17071147

**Published:** 2025-03-28

**Authors:** Hans F. Staender, Ewan Andrew Langan

**Affiliations:** 1 Clinic of Dermatology, Allergology and Venerology, University of Lübeck, 23560 Lübeck, Germany; hans.staender@student.uni-luebeck.de; 2 Department of Dermatological Sciences, University of Manchester, Manchester M13 9PL, UK

**Keywords:** cancer, melanoma, Real World Data, fixed dose, immunotherapy

## Abstract

Changes in the dosing schedules for immune checkpoint inhibitors, specifically nivolumab and pembrolizumab, in the treatment of metastatic melanoma, were introduced based on pharmacokinetic data and analysis of pre-existing clinical trial data in the absence of new clinical trials. Therefore, we sought to provide real-world data examining whether fixed-dose therapy (FDT) or weight-adapted therapy (WAT) influenced progression-free (PFS) and overall survival (OS), and the incidence of immune-related adverse events (irAEs). The electronic case notes of all patients (n = 77) treated with immune checkpoint inhibitor immunotherapy (ICI) in the first-line setting for melanoma in the Department of Dermatology, University of Luebeck, between the 1 January 2017 and the 31 December 2020, were retrospectively analysed. Although a higher proportion of patients in the WAT cohort were treated in the palliative setting, there were no correlations between dosing schedule, renal function, or BMI and PFS. Moreover, there were no differences between the cohorts in terms of PFS, OS, or the number and nature of irAEs. An elevated serum S100 concentration was associated with a decreased mean PFS in the FDT cohort (*p* < 0.001). This study, although inherently limited by its retrospective and monocentric nature, provides reassuring evidence that dosing schedule and pre-existing comorbidities do not influence efficacy or the irAE profile of ICI therapy in the management of melanoma.

## Simple Summary

Whilst immune checkpoint inhibition therapy has transformed the treatment of metastatic melanoma, there remains a lack of real-world data examining the potential effects of dosing schedule (weight-adapted versus fixed-dose schedule) on their efficacy and the development of immune-related adverse events (irAEs). In a monocentric retrospective analysis, we found no evidence that dosing schedule impacted treatment response or the development of irAEs in patients with melanoma. These observations should be confirmed in larger, register-based studies, but provide limited support for the potential of weight-adapted dosing.

## 1. Introduction

The treatment landscape of metastatic melanoma has been altered dramatically with the advent of immune checkpoint inhibition (ICI) therapy [1]. Anti-programmed cell death protein 1 (PD-1) and anti-cytotoxic T-lymphocyte-associated protein 4 (anti-CTLA4) antibodies are among the mainstays of ICI treatment. The recently published data from the seminal CheckMate 067 clinical trial have demonstrated median melanoma-specific survival of more than 120 months for combined ICI therapy (nivolumab (PD1 inhibitor)) plus ipilimumab (CTLA4 inhibitor) and 50 months for PD-1 inhibitor-based monotherapy [2]. In fact, almost 40% of patients in the combined ICI treatment arm were still alive after a decade. The main risk of treatment is the development of potentially irreversible, and in some cases fatal, immune-related adverse events (irAEs) [3]. Real-world data have confirmed that combined ICI therapy is associated with severe (grades 3 and 4) irAEs in over 50% of patients [4].

Unfortunately, between 40 and 65% of patients treated with anti-PD1 immune checkpoint therapy display primary resistance to treatment, whilst others develop resistance despite having initially responded to treatment [5,6]. The reasons behind the development of primary and secondary resistance are poorly understood. It is at least conceivable that treatment dose and/or dosing interval may impact treatment efficacy and the development of irAEs. For example, six weekly fixed-dose pembrolizumab administration was reportedly associated with an increased incidence of irDiabetes [7].

PD-1-based ICI is now routinely administered in a fixed-dose treatment schedule (FDT), irrespective of a patient’s body weight, although it was initially administered in a weight-adapted treatment (WAT) manner. Nivolumab and pembrolizumab were originally dosed according to body weight (3 mg/kg every two weeks and 2 mg/kg every three weeks, respectively). Both are now administered at fixed doses (nivolumab 240 mg fortnightly or 480 mg monthly, pembrolizumab 200 mg every 3 weeks or 400 mg every 6 weeks). FDT helps standardise treatment protocols, simplifying administration and potentially reducing the likelihood of dosage errors. The move from WAT to FDT has raised a question over whether the treatment dose and/or dosing interval may impact treatment efficacy and the development of irAEs.

Data from clinical trials and pharmacokinetic studies have found no evidence of dose- or interval-dependent differences in treatment efficacy [8,9,10,11]. However, real-world data examining the effects of FDT versus WAT remains relatively sparse. This is somewhat surprising, given that body mass index, kidney disease, and the development of immune-related adverse events have all been shown to affect the response to treatment to some extent [12,13,14]. To this end, we examined the effect of dosing schedule and patient- and melanoma-specific factors on treatment efficacy and irAEs in a retrospective monocentric study.

## 2. Materials and Methods

**Figure 1:**
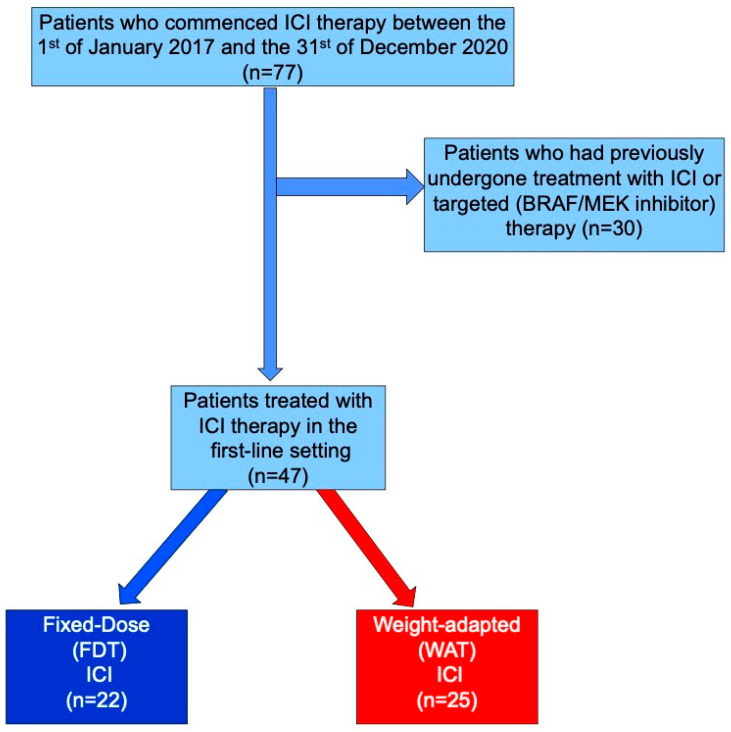
Flow chart of the study population.

Ethical approval was obtained from the Ethics Committee of the University of Luebeck (AZ 20-386, see attachment). The electronic case notes of all patients (n = 77) in whom treatment with immunotherapy was initiated in the Department of Dermatology of the University clinic Schleswig-Holstein, Campus Lübeck, for melanoma between the 1st of January 2017 and the 31st of December 2020 were retrospectively analysed. Thirty patients were excluded from further analyses, given that they did not receive treatment in the first-line setting. These patients had either undergone previous treatment with BRAF/MEK inhibitor-based targeted therapy or ICI-based immunotherapy, and their inclusion would have been a potential source of bias (Figure 1). Treatment response (progression-free survival (PFS)) was based on radiological evaluation according to RECIST (v1.1) criteria. PFS was measured in days from the date of treatment initiation to the first evidence of radiological progress. Overall survival (OS) was measured in days from the date of treatment initiation until death or the last day of follow-up (31.12.2020), whichever came sooner. It should be borne in mind that OS was undoubtedly influenced by subsequent lines of treatment, including surgery, ICI therapy (subsequent anti-PD1 monotherapy and/or combined anti-PD1 and anti-CTLA4 treatment), BRAF/MEK inhibitor therapy, or chemotherapy (dacarbazine) [15]. irAEs were classified according to the common terminology criteria for adverse events (CTCAE) version 5. All data were collated, anonymised, and analysed according to the Declaration of Helsinki principles. Laboratory values, including renal function, were recorded at baseline.

## 3. Statistical Analysis

Data were collected and integrated into Microsoft Excel (Version 16.82). All statistical analyses were performed using Microsoft Excel, and survival analyses were calculated using GraphPad Prism (version 8). Statistical advice was obtained from the Institute of Biomedical Statistics, University of Luebeck. Data are expressed as median, range, standard deviation, and 95% confidence intervals. *p* values of <0.05 were considered significant. Univariate analysis was used to determine the influence of variables on median overall survival (Log-Rank method). Data were analysed using Chi-Squared tests (categorical variables), or Kruskal–Wallis or Mann–Whitney U tests (continuous variables), depending on the distribution of the data.

## 4. Results

Between the 1 January 2017 and the 31 December 2020, a total of 77 patients received ICI therapy for melanoma at the out-patient unit of the Department of Dermatology and underwent regular clinical examination and laboratory investigation. Among them, 47 patients (females 15, males 32) underwent treatment with ICI therapy in the first-line treatment setting. The patient-, tumour- and treatment-related factors are shown in Table 1. The patients were categorised according to whether their initial ICI treatment was FDT (n = 25) or WAT (n = 22). The mean ages of the patients were 67.24 years (SD ± 16.98) in the FDT cohort and 74.82 years (SD ± 16.11) in the WAT cohort, which are not statistically different. Although the treatment setting was similar in both cohorts, a higher percentage of patients in the WAT cohort were treated in the palliative setting. However, there was no significant difference in ECOG performance status between the cohorts (Fisher’s exact test, *p* = 0.17). There were no significant differences in the numbers of patients receiving treatment with nivolumab (n = 25) or pembrolizumab (n = 22). (Table 1).

There was no significant difference between the groups in terms of renal function. The mean serum creatinine (±SD) levels were 84.41 ± 18.46 µmol/l and 101.7 ± 30.31 µmol/l in the FDT and WAT cohorts, respectively. The mean BMIs were 26.76 ± 4.45 and 27.47 ± 3.93 in the FDT and WAT cohorts, respectively. BMI was inversely correlated with PFS, but this correlation was not significant (*r* = −0.17 *p* = 0.25).

**Figure 2:**
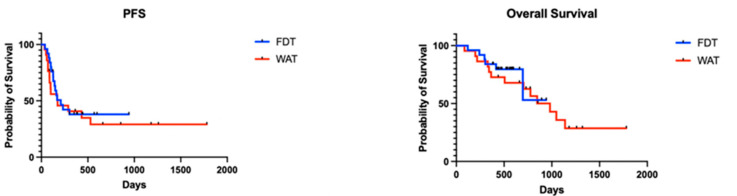
Progression-free and overall survival. There were no significant differences between the FDT and WAT cohorts.

There was no significant effect of the dosing modality (WAT versus FDT) on PFS. The median PFS was 208 days in the FDT group, compared to 174 days in the WAT group (Log-Rank Mantel-Cox Test, *p* = 0.595). Similarly, there was no significant difference in OS between the groups. The median OS durations were undefined and 980 days in the FDT and WAT cohorts, respectively (Log-Rank Mantel-Cox Test, *p* = 0.502). (Figure 2).

The potential influences of factors associated with response to ICI therapy were also investigated. The patient-specific factors were age [16,17], sex [18,19], body mass index [13,20,21], diabetes mellitus, and cardiovascular disease [22,23,24,25]. The melanoma tumour-specific factors encompassed BRAF status and serum S100 concentrations [26,27,28,29,30]. Finally, bearing in the mind the positive impact of the development of irAEs on treatment response to ICI, the development of irAEs was examined as a treatment-specific factor [12,31,32,33].

**Figure 3:**
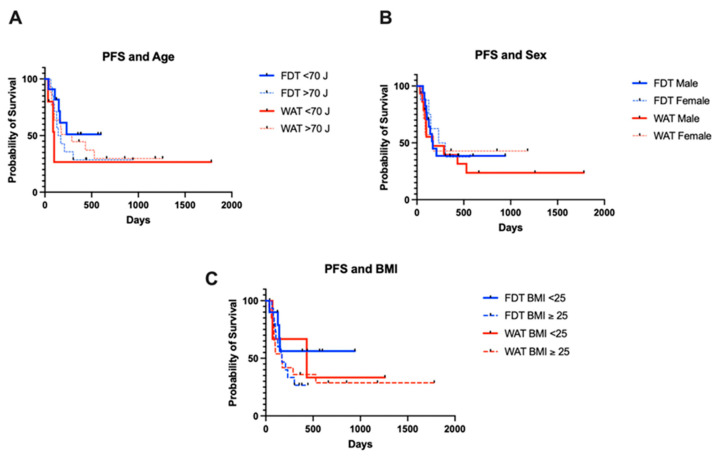
Patient-specific factors and progression-free survival. Age (**A**), sex (**B**) and body mass index (**C**) had no significant effects on PFS in either cohort.

The median PFS in patients under 70 years was not met in the FDT group and was 98 days in the WAT group. The equivalent figures were 155.5 and 288 days in patients 70 years and over in the FDT and WAT groups, respectively, with no significant differences between the groups. Similarly, there were no statistical differences in PFS between the groups according to sex or body mass index (Figure 3A–C). In terms of co-morbidities, there was also no significant difference in PFS depending on the presence of diabetes or coronary heart disease.

**Figure 4:**
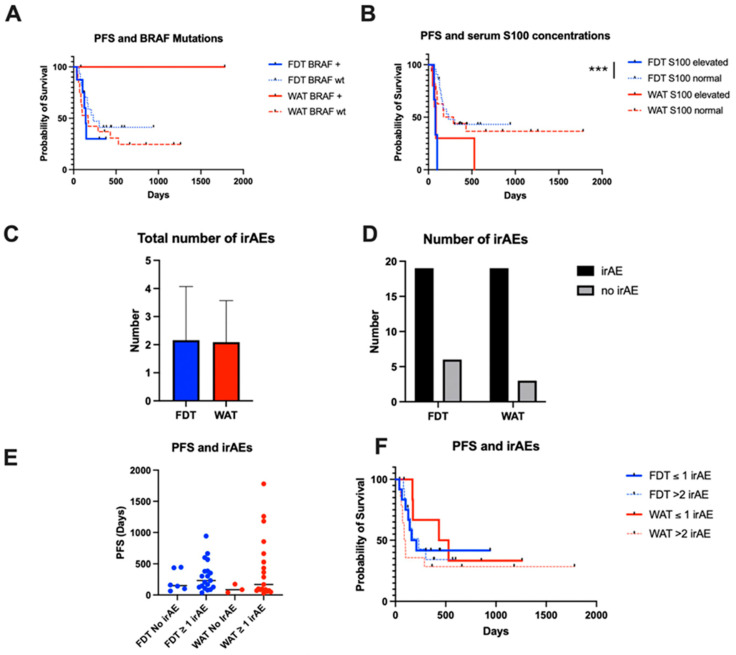
Disease-specific factors and irAEs. Whilst BRAF status had no effect on PFS (**A**), an elevated S100 concentration had a significant effect on PFS in the FDT group (**B**). There were no differences in the incidence or number of irAEs (**C**,**D**) between the cohorts, and the number of irAEs did not impact PFS (**E**,**F**).

Furthermore, whilst the presence of a BRAF mutation did not affect PFS in either group, an elevated S100 concentration was associated with significantly worse PFS in the FDT group (*p* < 0.001). Patients in the FDT cohort with an elevated S100 concentration had a median PFS of 79 days, compared to 232 days when S100 levels were within normal limits. In the WAT cohort, the median PFS was not significantly different, at 86 days when S100 concentration was elevated, compared to 288 days when S100 concentration was normal (*p* = 0.14). (Figure 4A,B).

Finally, the incidence of irAEs was determined between the groups, in addition to their potential influence on PFS. There were no differences in the overall number or incidence of irAEs between the cohorts and no significant effects of the incidence or number of irAEs on PFS. (Figure 4C–E).

## 5. Discussion

Although ipilimumab was first licensed in the European Union over a decade ago, shortly followed by pembrolizumab and nivolumab, their use continues to dramatically improve the treatment landscape for patients with locally advanced and/or metastatic melanoma in both the adjuvant and palliative treatment settings [34].

Based on pharmacokinetic data and flat exposure–response relationships for efficacy and safety, nivolumab fixed dosing was introduced without the need for additional clinical trials [35,36,37]. Similar data for pembrolizumab suggested that weight-adapted dosing offered no advantage over fixed dosing [8]. Modelling and simulation data have also supported the equivalence of pembrolizumab 400 mg every 6 weeks to 200 mg and 2 mg/kg every three weeks across various tumour types [38].

Here, there was no evidence that the use of FDT or WAT influenced either PFS or OS. These data support the available pharmacokinetic data that led to the change from WAT to FDT for both pembrolizumab and nivolumab [8,11,35,37,38]. Given that the WAT cohort had significantly worse renal function at the outset of treatment, coupled with a higher proportion of patients being treated in the palliative context, the lack of difference in PFS and OS between the cohorts is even more reassuring.

Indeed, the safety and efficacy of immune checkpoint inhibitors in patients with end-stage renal failure and dialysis have not revealed any marked differences in the incidence of irAEs or treatment response [39,40]. Consistent with the literature, there was no significant correlation between renal function and PFS or OS in either cohort. This provides reassuring real-world data that chronic renal impairment does not impact the efficacy of anit-PD1-based immune checkpoint inhibition, regardless of whether the treatment is given in a weight-adapted or fixed-dose manner.

We found no significant difference between the FDT and WAT groups in terms of average BMI. BMI was not correlated with treatment response in terms of PFS in the cohort as a whole, nor when the FDT and WAT groups were analysed separately. Moreover, BMI was not correlated with overall survival. It is conceivable that weight itself, rather than BMI, may influence treatment efficacy and the incidence of irAEs. However, Le Brun et al. [41]. did not find any evidence to support this in a nationwide retrospective study of almost 350 patients in France. In our study, there was no significant difference in BMI between the WAT and FDT groups. Whilst the present study was too small to examine the effects of body weight on treatment efficacy, it was reassuring that BMI was not associated with differences in irAEs or treatment responses.

The association between treatment response to immune checkpoint inhibitor therapy and body mass index remains controversial. Initial reports suggested that BMI correlated with an improved response to immune checkpoint therapy [13]. in males. This study was a retrospective multi-cohort analysis of over 2000 patients treated over a decade and included patients undergoing immune checkpoint, targeted and chemotherapy treatments. The association between obesity and improved PFS and OS was reported in men treated with immune checkpoint or targeted therapy for metastatic melanoma. Moreover, this association has been replicated across a range of solid tumour entities, including non-small-cell lung cancer and Merkel cell carcinoma, and in meta-analyses [42,43,44]. The findings are also indirectly supported by the increased incidence of irAEs in patients with obesity, often a mark of treatment response [45,46,47]. Ultimately, these findings led to the term the “obesity paradox” [48,49,50]. Whilst the underlying mechanisms are unclear, adipokines, including leptin, oxidative phosphorylation, a hypoxic tumour microenvironment, and the gastrointestinal microbiome have all been postulated to play a role [49,50,51,52]. However, recent studies and meta-analyses have cast doubt over the association between obesity and treatment response to immune checkpoint inhibitors per se, and point to a more nuanced understanding of the relationship between body weight and treatment response mediated through systemic inflammation, sarcopenia, subcutaneous or visceral adipose tissue distribution, and even whether the immune checkpoint treatment was administered in the first, or subsequent, lines of therapy [53,54,55,56,57].

There was a significant difference in PFS in the FDT cohort according to whether patients had elevated or normal serum S100 concentrations. This is in keeping with the prognostic value of S100 concentration in metastatic melanoma and response to immune checkpoint inhibition treatment [58,59,60,61]. It should be borne in mind that S100 is a less reliable marker of disease recurrence in the adjuvant setting, and the absolute number of patients with an elevated S100 concentration in the FDT group was particularly low, which may have skewed the result [62]. In any case, there was no effect of FDT versus WAT on PFS according to serum S100 concentrations or BRAF status. Again, this result comes with the caveat that patients with prior BRAF inhibitor therapy were excluded, mirrored by the overall low prevalence of BRAF mutations in the cohort.

Clinically, there has been a move towards up-front immune checkpoint therapy in BRAF-mutated patients, partially based on results from the CheckMate 067 trial [63]. which reported median PFS durations of 16.8 and 11.2 months in BRAF-mutated and BRAF wild-type patients, respectively. Furthermore, combined immune checkpoint immunotherapy (anti-PD1 and anti-CTLA4) resulted in superior overall response rates when administered up front, as compared to use in the second-line setting [64,65].

Turning towards irAEs, dermatological and endocrinological irAEs were among the most frequent, as reported in the literature [66,67]. There was no difference in the total number of irAEs between the FDT and WAT groups, and the incidence of irAEs did not translate into significant differences in PFS. In fact, the most recent meta-analysis and systematic review of irAEs in over 40,000 patients being treated for a range of tumour entities only confirmed mild correlations between immune checkpoint therapy effects on irAE rates and OS [68]. Moreover, the effect of irAEs on OS may be strongest, depending on both the affected organ system and tumour type, for example, cutaneous irAEs in the treatment of melanoma with immune checkpoint inhibitors [69].

Whilst irAEs seem to correlate with OS, depending on their number and severity, which organ system is affected, treatment (anti-PD1 monotherapy versus combined anti-PD1 and anti-CTLA4 treatment) and tumour type, this study only examined PFS and was not large enough to provide conclusive data on the effects of irAEs on OS. However, it is reassuring that neither FDT nor WAT, even in an adjuvant setting, resulted in significantly more irAEs.

The main strength of this study was the real-world nature of the data. There was no pre-selection of patients according to tumour burden, performance status, or co-morbidities, affording extrapolation of the data to routine clinical practice. Another key advantage was the availability of the full range of clinical parameters, from standard laboratory tests to mutational status, and corresponding treatment parameters, from treatment setting and number of treatment cycles to the nature and number of irAEs that were encountered. In contrast to meta-analyses and registry-based data, the full range of patient-, disease-, and treatment-specific factors were available for analysis. This allowed internal validation of the PFS data with the incidence of irAEs to provide robust and convincing data that neither FDT nor WAT were associated with significant differences in efficacy as measured by PFS, OS, or tolerability as measured by irAEs. The effects of treatment were also assessed in the first-line setting. These data also sit well with other recent reports, regarding the lack of effect of dosing schedules on patient outcomes [37,70], and confirms the utility of monitoring serum S100 concentrations during immune checkpoint treatment [26,58,60].

Limitations include the over-representation of male patients in both the FDT and WAT cohorts. It is also important to acknowledge that patients treated in both the palliative and adjuvant settings were analysed together. This limitation meant that both disease progression and recurrence were recorded together. However, there were no differences in age or performance status between the FDT and WAT cohorts, allowing comparisons to be made. The number of patients was also a limitation in this study, although this was unavoidable due to its monocentric nature and the exclusion of treatment in the second-line setting to avoid a potentially confounding variable. Another potential limitation was the lack of randomisation due to its retrospective nature. This was a retrospective analysis, before and after the change in the licencing of nivolumab and pembrolizumab from weight-adapted to fixed dosing. Therefore, all drugs were administered in label according to the product license at the time. Despite the limitations, it is reassuring to report the lack of differences in efficacy, supported by similar levels of irAEs, between the groups. This finding supports the pharmacokinetic and limited recently reported real-world data [70].

In the real-world setting, across both palliative and adjuvant treatment settings, there was no evidence of differences between the overall response rates of patients with metastatic or locally advanced melanoma to immune checkpoint inhibitor therapy based on the dosing schedule [71]. Patients in the FDT and WAT cohorts had similar median PFS and experienced a similar profile and number of irAEs. Moreover, immune checkpoint inhibitor efficacy was not influenced by impaired renal function or body mass index. Finally, patient- and treatment-specific factors did not affect treatment response in either group. As expected, increased serum S100 concentrations were associated with a poorer response to treatment, as was seen in the FDT cohort. Although this difference was not observed in the WAT group, there was no difference overall between the groups in terms of PFS according to S100 concentrations. Given that FDT was introduced in 2018, the period of follow-up was not the same between the groups in terms of OS. However, there was no indication that OS differed over the period of this study. Prospective multi-centric trials are needed to confirm the findings reported here, specifically in patients with moderate- and/or high-risk obesity (BMI ≥ 35), who are under-represented here. Moreover, multivariate analyses of the efficacy parameters should be considered in larger, register-based studies.

## 6. Conclusions

The data herein support the existing pharmacokinetic and recently published real-world data suggesting that FDT delivers comparable efficacy and tolerability to WAT, whilst potentially delivering significant health care savings in the context of an increasingly overweight general population [41,70]. On the other hand, there may be cases where WAT may provide potential cost savings, should this option be available for underweight patients.

## Figures and Tables

**Table 1:** Baseline characteristics and demographics in the cohorts.

	Fixed Dose Therapy (FDT)	Weight Adapted Therapy (WAT)
Total number of patients		
Number	25	22
Sex		
Male	17	15
Female	8	7
Age (Years)		
<70	11	5
≥70	14	17
Mutation status		
BRAF	8	2
NRAS	9	9
cKit	1	2
ECOG Status		
0	14	19
1	10	3
2	1	0
Melanoma Stage		
IIc	0	2
III	16	10
IV	9	10
S100 elevated		
Yes	3	5
no	22	17
Cardiovascular Disease		
Yes	10	10
No	15	12
Diabetes		
Yes	7	6
no	18	16
Boby Mass Index		
<25	10	4
≥25	15	18

## Data Availability

The original contributions presented in this study are included in the article. Further inquiries can be directed to the corresponding authors.
